# Metal−Organic Frameworks Nucleated by Silk Fibroin and Modified with Tumor‐Targeting Peptides for Targeted Multimodal Cancer Therapy

**DOI:** 10.1002/advs.202302700

**Published:** 2023-08-23

**Authors:** Yuping Chen, Ruyin Lyu, Jie Wang, Qichao Cheng, Yanfang Yu, Shuxu Yang, Chuanbin Mao, Mingying Yang

**Affiliations:** ^1^ Institute of Applied Bioresource Research College of Animal Science Zhejiang University Yuhangtang Road 866 Hangzhou Zhejiang 310058 P. R. China; ^2^ Jiangxi Cash Crops Institute Nanchang Jiangxi 330202 P. R. China; ^3^ Department of Neurosurgery Sir Run Run Shaw Hospital School of Medicine Zhejiang University 3 East Qingchun Road Hangzhou Zhejiang 310016 P. R. China; ^4^ Department of Biomedical Engineering The Chinese University of Hong Kong Sha Tin Hong Kong SAR P. R. China; ^5^ School of Materials Science & Engineering Zhejiang University Hangzhou 310027 China

**Keywords:** *Bombyx mori* (*B. mori*) silk fibroin, drug carrier, triple therapeutic effects, tumor‐targeting peptide, zeolitic imidazolate framework‐8 (ZIF‐8)

## Abstract

Multimodal therapy requires effective drug carriers that can deliver multiple drugs to specific locations in a controlled manner. Here, the study presents a novel nanoplatform constructed using zeolitic imidazolate framework‐8 (**Z**IF‐8), a nanoscale metal‐organic framework nucleated under the mediation of silk fibroin (**S**F). The nanoplatform is modified with the newly discovered MCF‐7 breast tumor‐targeting peptide, AREYGTRFSLIGGYR (**AR** peptide). Indocyanine green (**I**CG) and doxorubicin (**D**OX) are loaded onto the nanoplatform with high drug encapsulation efficiency (>95%). ICG enables the resultant nanoparticles (NPs), called AR‐ZS/ID‐P, to release reactive oxygen species for photodynamic therapy (**P**DT) and heat for photothermal therapy (**P**TT) under near‐infrared (NIR) irradiation, promoting NIR fluorescence and thermal imaging to guide DOX‐induced chemotherapy. Additionally, the controlled release of both ICG and DOX at acidic tumor conditions due to the dissolution of ZIF‐8 provides a drug‐targeting mechanism in addition to the AR peptide. When intravenously injected, AR‐ZS/ID‐P NPs specifically target breast tumors and exhibit higher anticancer efficacy than other groups through ICG‐enabled PDT and PTT and DOX‐derived chemotherapy, without inducing side effects. The results demonstrate that AR‐ZS/ID‐P NPs are a promising multimodal theranostic nanoplatform with maximal therapeutic efficacy and minimal side effects for targeted and controllable drug delivery.

## Graphical abstract

A novel multi‐functional nanoplatform is developed from ZIF‐8 nucleated and grown on silk fibroin, presenting high drug encapsulation efficiency for both ICG and DOX. Being modified with tumor‐affinity peptide, the constructed nanoplatform targets the tumor sites and releases drugs in an acidic microenvironment, achieving chemo/photodynamic/photothermal triple‐modal therapeutic outcomes under the guidance of two‐way imaging.

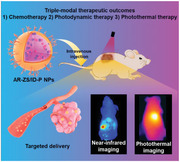

## 1 Introduction

Phototherapy, consisting of photodynamic therapy (PDT) and photothermal therapy (PTT), has emerged as a promising noninvasive and effective approach for cancer treatment.^[^ ^1^ ^]^ Indocyanine green (ICG) is an ideal photosensitizer for PDT/PTT due to its response to the tissue‐penetrating infrared irradiation.^[^ ^2^ ^]^ Importantly, ICG can also be used for thermal imaging and near‐infrared fluorescence imaging to achieve image‐guided tumor treatment.^[^ ^3^ ^]^ Tumor imaging provides crucial information for the diagnosis, staging, treatment planning, and monitoring of tumors.^[^ ^4^ ^]^ However, the hydrophilic nature of free ICG in vivo poses challenges such as a short half‐life and poor cellular uptake.^[^ ^5^ ^]^ To achieve high spatiotemporal accuracy of PDT/PTT, it is crucial to design a smart drug carrier that can load ICG and meet requirements such as stimuli‐responsive release and tumor‐targeting.^[^ ^6^ ^]^ Additionally, the carrier should be able to load and sustain chemotherapeutic drugs to enhance the synergistic effect of chemotherapy and PDT/PTT for improved therapeutic outcomes.

Zeolitic imidazolate framework‐8 (ZIF‐8), a subclass of metal−organic frameworks (MOFs), offers a viable solution for achieving controllable drug delivery.^[^ ^7^ ^]^ ZIF‐8 exhibits unique characteristics, such as controllable synthesis, good chemical stability, and pH‐sensitive degradation properties.^[^ ^8^ ^]^ Furthermore, the size of ZIF‐8 can easily be controlled below 150 nm, which facilitates the “enhanced permeability and retention” (EPR) effects, driving particles to target tumors and making ZIF‐8 a promising candidate for efficient, stimuli‐responsive drug release.^[^ ^9^ ^]^ Biomimetic mineralization is a simple method to synthesize nanoscale ZIF‐8, as it allows for precise control of the pore shape,^[^ ^10^ ^]^ chemical functionality,^[^ ^11^ ^]^ and size.^[^ ^12^ ^]^ Therefore, identifying a suitable template to induce mineralization of nanoscale ZIF‐8 with a size of less than 150 nm is crucial.

Biomacromolecules, such as proteins, DNA, and enzymes, have been reported to serve as bio‐templates for synthesizing nanoscale ZIF‐8.^[^ ^13^ ^]^ *Bombyx mori* (*B. mori*) silk fibroin (SF) is a bio‐template that offers abundant carboxyl groups as nucleation sites.^[^ ^14^ ^]^ Our team has successfully used SF as a bio‐template to mediate the nucleation and deposition of apatite crystals^[^ ^15^ ^]^ and silica^[^ ^16^ ^]^ in nanoscales. Additionally, we confirmed the formation of ZIF‐8 on electrospun SF nanofibers,^[^ ^17^ ^]^ making it possible to prepare nanoscale ZIF‐8 with size controlled at 100 nm using SF as a bio‐template. Moreover, SF itself can be utilized as a drug carrier due to its excellent properties of biocompatibility, biodegradability, and low immunogenicity.^[^ ^18^ ^]^ The hydrophilic terminal blocks at both the amino and carboxy‐terminal of SF provide favorable adsorption sites for drugs, leading to a higher drug‐loading rate.^[^ ^19^ ^]^ Hence, using SF as a bio‐template to trigger the nucleation and deposition of nanoscale ZIF‐8 as a drug carrier is a rational approach.

In addition, to enhance treatment efficiency and reduce side effects, it is desirable to deliver target drug molecules to specific tumor sites.^[^ ^20^ ^]^ Tumor‐recognizing molecules such as antibodies,^[^ ^21^ ^]^ folic acid/folate,^[^ ^22^ ^]^ and hyaluronic acid^[^ ^23^ ^]^ have been used for targeted drug delivery. However, these molecules primarily interact with certain types of proteins that are overexpressed or explicitly expressed at the tumor sites, neglecting patient‐specific tumor differences for the same type of cancer.^[^ ^24^ ^]^ To overcome this challenge, we utilized in vivo phage display to identify a tumor‐targeting peptide, AREYGTRFSLIGGYR (termed as AR), which actively targets MCF‐7 tumors.^[^ ^25^ ^]^ We plan to use AR to guide nanoparticles (NPs) for precision medicine in MCF‐7 breast cancer therapy.

**Scheme 1:**
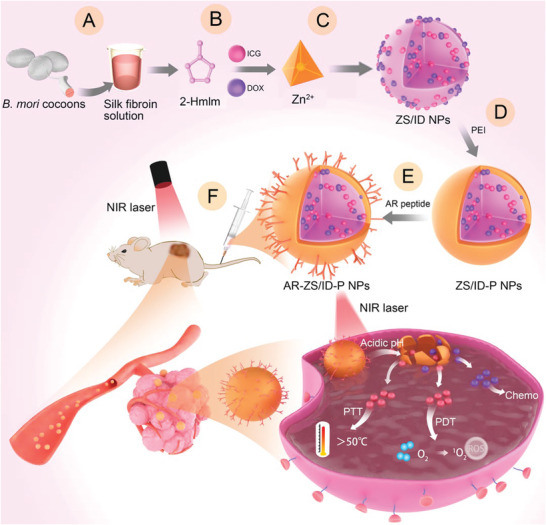
Schematic illustration for the preparation of AR‐ZS/ID‐P NPs and target peptide‐guided breast cancer therapy. A–C) The silk fibroin (SF) was prepared and added to the 2‐HmIm aqueous solution to serve as a bio‐template (A), followed by the addition of the anticancer drug DOX, the photosensitizer ICG (B), and then the Zn(NO_3_)_2_·6H_2_O aqueous solution to form drug‐loaded ZIF‐8 nanoparticles (termed ZS/ID NPs) by one‐pot process (C). D) A PEI coating strategy was introduced to ZS/ID NPs (termed ZS/ID‐P NPs) to improve the stability of nanoparticles and provide rich binding sites for MCF‐7 breast tumor‐targeting peptides. E) The MCF‐7 breast tumor‐targeting peptide AR was linked with ZS/ID‐P NPs (termed AR‐ZS/ID‐P NPs) under the action of EDC/NHS crosslinkers. F) AR‐ZS/ID‐P NPs injected into the caudal vein of mice were guided to tumor by the AR peptide, allowing the NPs to selectively accumulate within the tumor. Then, in response to the acidic environment in cancer cells, AR‐ZS/ID‐P NPs were dissolved. Thus DOX and ICG were released from the AR‐ZS/ID‐P NPs into cancer cells, leading to cancer cell death with the help of an additional 808 nm laser.

Hence, we have designed a nanoscale ZIF‐8 that is pH‐sensitive and targets tumors for synergistic cancer therapy using chemo/PDT/PTT (**Scheme** **1**). First, we prepared and added SF to the 2‐Methylimidazole (2‐HmIm) aqueous solution to serve as a bio‐template (Scheme 1A). Next, we co‐cultured the anticancer drug DOX and photosensitizer ICG with the mixed solution (Scheme 1B), followed by the rapid induction of nucleation and formation of DOX/ICG‐loaded nanoscale ZIF‐8 (termed as ZS/ID NPs) by a one‐pot process with the addition of Zn^2+^ (Scheme 1C). To achieve long‐term stability and rich binding sites for the conjugation of MCF‐7 breast tumor‐targeting peptide, we introduced a polyethylenimine (PEI) coating onto ZS/ID NPs (ZS/ID‐P NPs) (Scheme 1D). Finally, we linked the MCF‐7 breast tumor‐targeting peptide AR with the PEI on the ZS/ID‐P NPs (termed AR‐ZS/ID‐P NPs) to selectively inhibit the breast tumor (Scheme 1E,F). In this way, AR‐ZS/ID‐P NPs exhibit tumor‐homing capabilities due to the tumoral pH‐responsive and peptide‐targeting properties and can realize chemo/PDT/PTT triple‐modal therapeutic outcomes. We found that AR‐ZS/ID‐P NPs largely accumulated in tumor tissue, achieved super‐additive antitumor efficacy, and reduced light‐associated side effects.

## 2 Results and Discussion

### 2.1 Characterization of Different Nanoparticles

**Figure 1:**
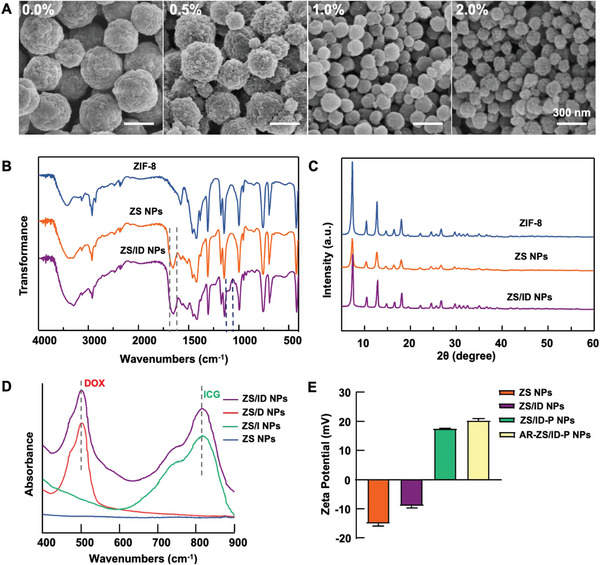
Characterization of different nanoparticles. A) SEM images of different ZS NPs synthesized using various mass fractions of SF (0%, 0.5%, 1.0%, and 2.0%) as a bio‐template. B) FTIR spectra and C) PXRD patterns of ZIF‐8, ZS NPs, and ZS/ID NPs. D) Absorption spectra of ZS NPs, ZS/I NPs, ZS/D NPs, and ZS/ID NPs. E) Zeta potential distribution of ZS NPs, ZS/ID NPs, ZS/ID‐P NPs, and AR‐ZS/ID‐P NPs.

To explore the contribution of SF in forming ZS NPs, we increased the concentration of SF from 0.0% to 2.0%. The SEM images showed that the size of ZS NPs decreased from about 400 nm to 80 nm, becoming more uniform and dispersed (**Figure** **1**A). Besides, SF not only impacted the size of ZS NPs but also influenced their crystallization process. The formation of ZS NPs (6 min) mediated by SF was faster than the formation of ZIF‐8 (≈8 min), owing to the abundant nucleation sites of SF that initiated the ZS NPs formation (Figure S1, Supporting Information). In addition, the loading of ICG and DOX (termed ID) affected the characteristics of ZS NPs, such as their size, homogeneity, and dispersibility. The size and uniformity of ZS/ID NPs reduced with the increase of ID concentration from 0.5 to 4.0 mg mL^−1^ (Figure S2, Supporting Information). Based on these findings, final concentrations of 1.0% SF and 2.0 mg mL^−1^ ID were selected to obtain ZS/ID NPs with desirable size (100–150 nm), uniformity, and dispersibility.

Further characterizations of different nanoparticles are depicted in Figure 1B–E. The fourier transform infrared spectroscopy (FTIR) spectra of ZS NPs and ZS/ID NPs presented distinct peaks at 1651 and 1519 cm^−1^, indicating the presence of SF's amide I and II peaks (Figure 1B). The powder X‐ray diffraction (PXRD) analysis revealed that ZS NPs and ZS/ID NPs maintained the same highly crystalline structure with ZIF‐8 (Figure 1C). These findings demonstrated that SF could act as a bio‐template for inducing the formation of ZS NPs, while retaining their crystal structure. The absorption spectra of ZS/ID NPs showed two absorption peaks that corresponded to DOX and ICG, respectively, indicating their successful loading into the ZS/ID‐NPs (Figure 1D). Moreover, the increase in zeta potential from the ZS NPs (−14.9 mV) to ZS/ID NPs (−8.88 mV) confirmed the successful loading of ID. Based on the dynamic light scattering (DLS) results, it was observed that the loading of the ID had no significant effect on the particle size of ZS NPs (approximately 120 nm). However, the introduction of AR peptide modification to ZS/ID NPs led to an increase in the particle size (around 135 nm) and enhanced the dispersibility of the nanoparticles in the solution. Notably, the lower polydispersity index (PDI) value indicated a narrower and more uniform size distribution (Figure S3 and Table S1, Supporting Information). The coating of PEI and modification of AR tumor‐targeting peptides were also verified by the change in their zeta potential (Figure 1E).

### 2.2 The Loading and Release Behaviors of AR‐ZS/ID‐P NPs

Figure S4 (Supporting Information) illustrated the effect of varying drug concentrations (0.5, 1.0, 2.0, and 4.0 mg mL^−1^) on the encapsulation efficiency (EE) of DOX and ICG in AR‐ZS/ID‐P NPs. The results showed that AR‐ZS/ID‐P NPs had a high EE for both DOX and ICG, with an EE approaching 100% when the drug concentration was greater than 2 mg mL^−1^ (Figure S4A, Supporting Information). It was clear that the drug encapsulation efficiency increased with the rise in drug concentration, which may be attributed to the adsorption and encapsulation effects and physical interactions.^[^ ^26^ ^]^ The release behavior of AR‐ZS/ID‐P NPs was studied in neutral (pH 7.4) and acidic PBS buffers (pH 6.4 and 5.0), as shown in Figure S4B (Supporting Information). Only 35% of DOX and 30% of ICG were released in the PBS buffer at pH 7.4 within 24 h, indicating that AR‐ZS/ID‐P NPs have a slow‐release ability in neutral conditions. Conversely, nearly 80% of DOX and 90% of ICG were released in the PBS buffers with pH at 5.0 and 6.4 within 24 h. Figure S5 (Supporting Information) shows the morphologies of AR‐ZS/ID‐P NPs after incubation in different pH solutions for 24 h. Under pH 7.4, the morphology of the AR‐ZS/ID‐P NPs remained almost unchanged. However, under pH 6.4 and 5.0, the AR‐ZS/ID‐P NPs were completely degraded, and no nanoparticles were observed. This suggested that DOX and ICG were preferentially released from AR‐ZS/ID‐P NPs in an acidic environment and their release was stimulated in the acidic tumor microenvironment. The pH‐responsive release behavior of AR‐ZS/ID‐P NPs might be due to the increased solubility of loaded DOX and ICG in acidic environments. The degradation of AR‐ZS/ID‐P NPs may be attributed to the acidic degradation characteristics of ZIF‐8. In acidic conditions, the protonation of imidazolate linkers can lead to the cleavage of metal‐ligand bonds, resulting in the degradation of the ZIF‐8 structure. Under neutral and alkaline conditions, the imidazolate linkers remain deprotonated, maintaining the integrity of the metal‐ligand coordination bonds and the stability of the ZIF‐8 structure.^[^ ^27^ ^]^

### 2.3 In vitro Photothermal and Photodynamic Abilities of the NPs

**Figure 2:**
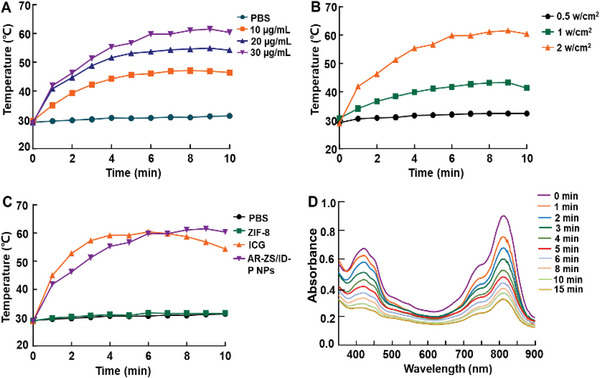
In vitro photothermal and photodynamic abilities of the NPs. A) Quantitative temperature changes of AR‐ZS/ID‐P NPs with different concentrations of ICG under 808 nm irradiation (2 W cm^−2^), PBS was set as a control. B) Quantitative temperature changes of AR‐ZS/ID‐P NPs (with an ICG concentration of 30 µg mL^−1^) under different laser power densities with the irradiation of 808 nm. C) Quantitative temperature changes of ICG (30 µg mL^−1^), AR‐ZS/ID‐P NPs (with an ICG concentration of 30 µg mL^−1^), ZIF‐8, and PBS under 808 nm irradiation (2 W cm^−2^). D) Normalized UV–vis absorption spectra of AR‐ZS/ID‐P NPs with DPBF single oxygen probe after irradiation with 808 nm laser (2 W cm^−2^), reflecting the ROS generation of AR‐ZS/ID‐P NPs.

**Figure** **2**A–C demonstrates the PTT efficacy of AR‐ZS/ID‐P NPs in the near‐infrared‐triggered (NIR). Laser intensity and ICG concentration played significant roles in the photothermal conversion capabilities of AR‐ZS/ID‐P NPs (Figure 2A,B). The photothermal response of AR‐ZS/ID‐P NPs was dependent on the ICG concentration. Under NIR irradiation maintained at 2 W cm^−2^, increasing the ICG concentration from 10 to 30 µg mL^−1^ led to an increase in the maximum temperature of AR‐ZS/ID‐P NPs from 42.1 to 60.3 °C (Figure 2A). Moreover, reducing the laser‐power density decreased the temperature increase rate. As depicted in Figure 2B, laser power reduction to 0.5 W cm^−2^ resulted in the maximum temperature of AR‐ZS/ID‐P NPs reaching only 31.2 °C.

Additionally, the photothermal conversion capabilities of AR‐ZS/ID‐P NPs, PBS, ZIF‐8, and free ICG were compared (Figure 2C). While PBS and ZIF‐8 showed negligible temperature increases, free ICG demonstrated similar photothermal conversion capabilities to AR‐ZS/ID‐P NPs. However, free ICG caused a decrease in temperature earlier than AR‐ZS/ID‐P NPs, indicating that AR‐ZS/ID‐P NPs enhanced the photostability of free ICG. Furthermore, by analyzing the temperature increment curve and the cooling curve of AR‐ZS/ID‐P NPs (Figure S6A, Supporting Information), a clear linear relationship between time and the cooling temperature after the photothermal transformation was obtained (Figure S6B, Supporting Information). Based on these findings, the photothermal conversion efficiency of AR‐ZS/ID‐P NPs was calculated to be 40.63%. Then, the ability of AR‐ZS/ID‐P NPs to generate ROS when irradiated by NIR laser was investigated. As depicted in Figure 2D, the absorption of 1,3‐Diphenylisobenzofuran (DPBF) in the AR‐ZS/ID‐P NPs solution showed a sustained decline upon prolonging the laser irradiation time, indicating the generation of ROS. These results indicated that AR‐ZS/ID‐P NPs can serve as therapeutic agents in NIR‐triggered PTT and PDT.

### 2.4 In Vitro Targeted Cancer Therapy

**Figure 3:**
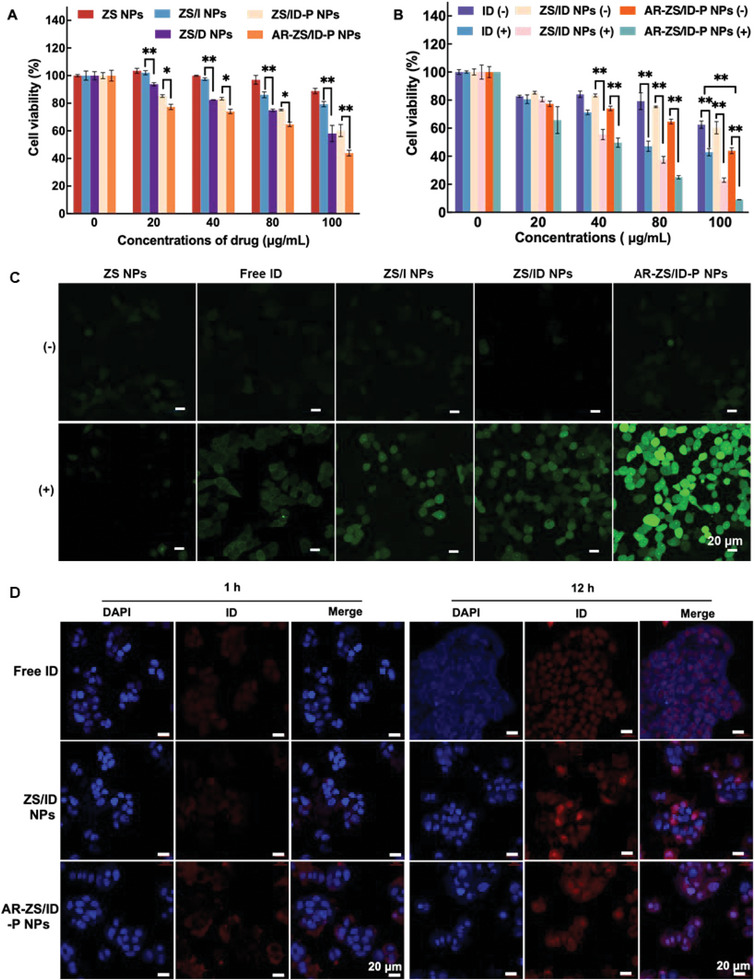
Cytotoxicity of different nanoparticles. A) Relative viability of MCF‐7 cancer cells exposed to different nanoparticles (ZS NPs, ZS/I NPs, ZS/D NPs, ZS/ID‐P NPs, and AR‐ZS/ID‐P NPs) with a series of concentrations for comparing their chemotherapy effects. B) Relative viability of MCF‐7 cells co‐cultured with different concentrations of nanoparticles under the 808 nm laser irradiation and without the laser irradiation, confirming the photodynamic/photothermal therapy of different samples. *p < 0.05; **p < 0.01. C) Intracellular ROS generation of MCF‐7 cells incubated with different treatments. (+): with 808 nm laser irradiation; (‐): without laser irradiation. D) The confocal fluorescence images of MCF‐7 cancer cells with different treatments (Free ID, ZS/ID NPs, and AR‐ZS/ID‐P NPs) for 1 h and 12 h. The cell nucleus was dyed with DAPI (blue).

**Figure** **3**A depicts the cytotoxicity of ZS NPs, ZS/I NPs, ZS/D NPs, ZS/ID NPs, and AR‐ZS/ID‐P NPs. Inappreciable cell death occurred in the groups of ZS NPs and ZS/I NPs even at a high concentration, indicating their good biocompatibility. The testing groups loaded with DOX exhibited dose‐dependent toxicity to MCF‐7 cells, likely due to the chemotherapy effect of DOX. Notably, the AR‐ZS/ID‐P NPs group resulted in about 50% cell death, while only 40% of cells died when incubated with ZS/D NPs and ZS/ID NPs. Figure 3B demonstrates the synergistic effects of chemotherapy and PDT/PTT from free ID, ZS/ID NPs, and AR‐ZS/ID‐P NPs on MCF‐7 cancer cells. All testing groups showed significantly enhanced toxicity to MCF‐7 cancer cells irradiated by 808 nm laser compared to the control group without laser irradiation. Fluorescence live/dead cell images of MCF‐7 cells also confirmed the excellent cytotoxicity of the AR‐ZS/ID‐P NPs under laser irradiation (Figure S7, Supporting Information). In order to validate the PDT effect of the samples, 2′,7′‐Dichlorodihydrofluorescein diacetate (DCFH‐DA) was employed as an intracellular ROS probe to observe intracellular ROS generation under different treatments. Consistent with the results of the CCK‐8 assay and live/dead staining, the 808 nm laser‐irradiated AR‐ZS/ID‐P NPs exhibited the highest capability of ROS generation (Figure 3C). This could be attributed to the targeting effect of AR peptide, which facilitated the uptake of AR‐ZS/ID‐P NPs by cancer cells and led to higher toxicity to cancer cells. Therefore, further investigations were conducted to explore the intracellular localization of different samples. Figure 3D displays the cellular uptake of free ID, ZS/ID NPs, and AR‐ZS/ID‐P NPs, which was consistent with the results shown in Figure 3A,B. The cells co‐cultured with AR‐ZS/ID‐P NPs displayed the strongest red fluorescence among all groups after 1 h of irradiation. After 12 h of incubation, more dead cells were observed in the AR‐ZS/ID‐P NPs group, which could be due to more AR‐ZS/ID‐P NPs homing on the MCF‐7 breast cancer cells, attributed to the targeting ability of the AR peptide, and subsequently killing the cells more effectively. The TEM images further confirmed that AR‐ZS/ID‐P NPs entered MCF‐7 breast cancer cells within 6 h (Figure S8, Supporting Information).

**Figure 4:**
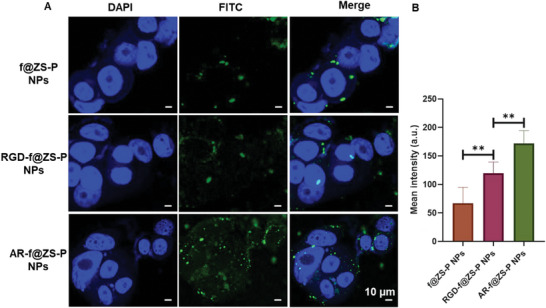
Targeting effect of AR‐f@ZS‐P NPs in vitro. A) The confocal fluorescence images and B) fluorescence intensity quantification analysis of the MCF‐7 cells co‐cultured with FITC‐labeled NPs (green), including f@ZS‐P NPs, RGD‐f@ZS‐P NPs, and AR‐f@ZS‐P NPs. DAPI (blue) was used for dying the cell nucleus.

**Figure** **4** further confirms the excellent tumor‐homing ability of AR peptide‐modified NPs. Compared with those groups treated by f@ZS‐P NPs and RGD‐f@ZS‐P NPs, the cells in the AR‐f@ZS‐P NPs group showed that the strongest fluorescence and the fluorescence was detected in the cytoplasm as well as the cell nucleus (Figure 4A). In contrast, nearly all NPs in the groups of f@ZS‐P NPs and RGD‐f@ZS‐P NPs existed in the cytoplasm. The fluorescence intensity quantification analysis also revealed that the green fluorescence signal in MCF‐7 cells was significantly enhanced in the AR‐f@ZS‐P NPs treated group compared to groups f@ZS‐P NPs and RGD‐f@ZS‐P NPs (Figure 4B). These results indicated that AR peptide enabled the NPs to target and recruit the MCF‐7 breast cancer cells.

### 2.5 In Vivo Tumor Targeting Ability of AR‐ZS/ID‐P NPs

**Figure 5:**
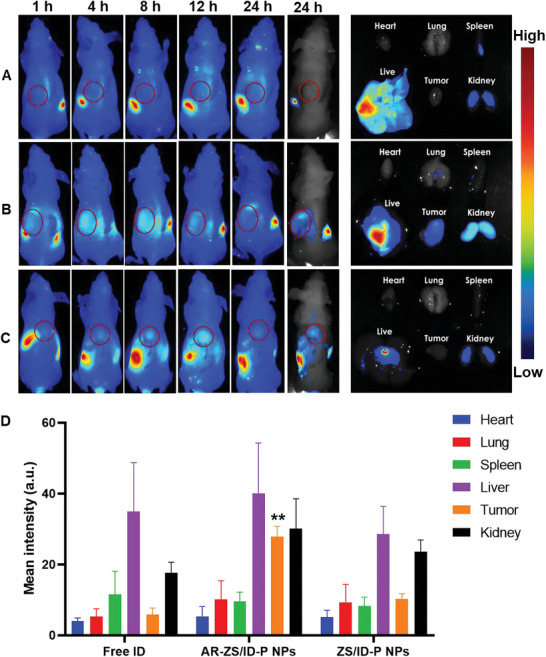
In vivo imaging for detection of tumor‐targeting ability of different samples at the MCF‐7 tumors. One hundred microliters of a sample solution, A) free ID, B) AR‐ZS/ID‐P NPs, or C) ZS/ID‐P NPs), with an equal ICG concentration of 60 µg mL^−1^ was injected into the caudal vein of corresponding mice. After injection, the images were acquired at 1, 4, 8, 12, and 24 h, the tumors were highlighted by red circles. After that, the tumor and other major organs (heart, lungs, spleen, kidney, and liver) were excised and their fluorescence images were recorded. All images shared the same parameters, so the intensity difference reflected the actual difference in the tumor‐targeting ability of samples. D) Quantitative analysis of the bioluminescence signal intensity in ex vivo tissues collected after 24 h.

The NIR fluorescence images illustrated the in vivo tumor‐specific accumulation of free ID, ZS/ID‐P NPs, and AR‐ZS/ID‐P NPs. Each group exhibited distinct biodistribution and tumor accumulation behaviors, as shown in **Figure** **5**. Free ID was rapidly metabolized and eliminated from the bloodstream, with negligible signal detected in the tumor site. Conversely, the mice treated with AR‐ZS/ID‐P NPs demonstrated clear tumor contrast just 1 h after intravenous injection, with the highest contrast signal observed after 4 h. More importantly, the high contrast signal was also detected at 24 h. In contrast, in the ZS/ID‐P NPs group, the highest contrast signal was found at 12 h, and only a weak signal was detected at 24 h. Moreover, the fluorescence signals of dissected organs confirmed the enhanced selectivity of AR‐ZS/ID‐P NPs for tumor tissues. Only the AR‐ZS/ID‐P NPs group displayed the fluorescence signal in the tumor site compared to free ID and ZS/ID‐P NPs (Figure 5A–C). The quantitative analysis of bioluminescence signal intensity in ex vivo tissues further confirmed the significantly enhanced fluorescence signal at the tumor site in the AR‐ZS/ID‐P NPs treated group (Figure 5D). These results confirmed that AR modification helped AR‐ZS/ID‐P NPs rapidly accumulate in the tumor and remain in the body for a relatively longer time.

**Figure 6:**
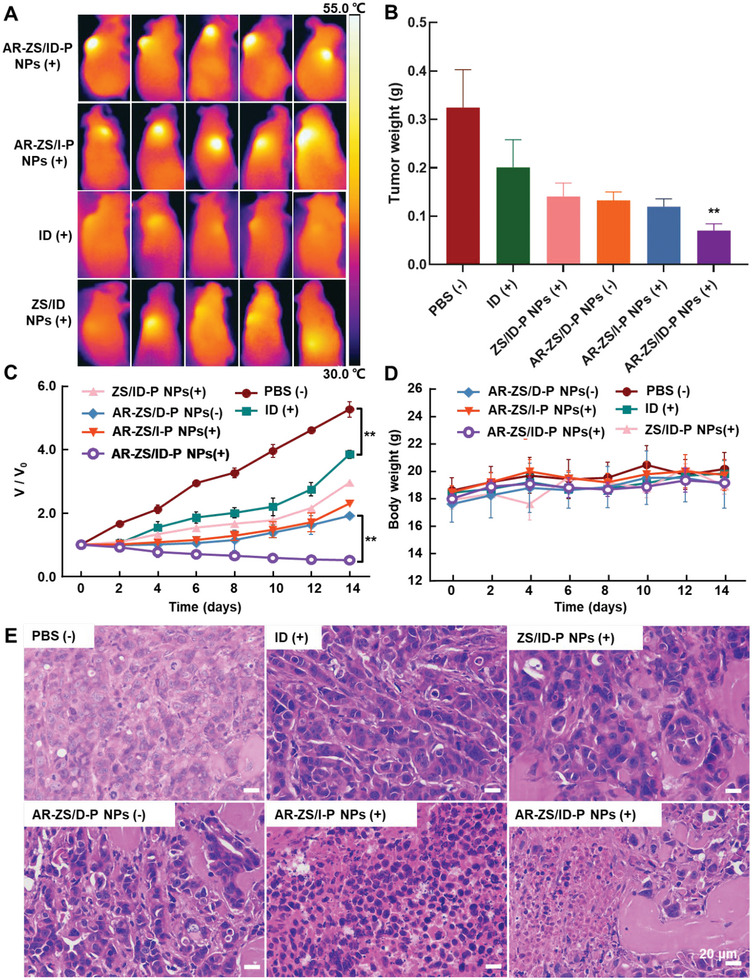
The in vivo tumor‐targeted breast cancer therapy by the AR‐ZS/ID‐P NPs + laser group via synergistic photothermal/photodynamic and chemotherapy. A) Thermal images of MCF‐7 tumor‐bearing mice injected with AR‐ZS/ID‐P NPs, AR‐ZS/I‐P NPs, ID, and ZS/ID NPs after 5 min 808 nm laser irradiation (2 W cm^−2^). B) Weight of tumors excised 14 days after the treatment of PBS, free ID + laser, ZS/ID‐P NPs + laser, AR‐ZS/D‐P NPs, AR‐ZS/I‐P NPs + laser, and AR‐ZS/ID‐P NPs + laser. C) Tumor volume and D) body weight change of mice after different treatments indicated. E) H&E‐staining of tumors excised from MCF‐7 tumor‐bearing mice receiving the indicated treatments. *p < 0.05; **p < 0.01. (+), under NIR light, (‐), in the absence of NIR light.

Combining NIR fluorescence and IR thermal imaging provided more precise information on the in vivo targeting ability of AR‐ZS/ID‐P NPs. Four hours post intravenous injection, the tumor surface temperature of the AR peptide‐modified groups, including the AR‐ZS/ID‐P NPs and AR‐ZS/I‐P NPs groups, rapidly rose to over 50 °C within 5 min of irradiation. In contrast, only weak thermal signals were observed in the free ID and ZS/ID NPs groups, indicating the minimal homing of ID or ZS/ID NPs to the tumor sites (**Figure** **6**A). Overall, we validated that the AR peptide improved NPs' homing to tumor sites.

### 2.6 In Vivo Antitumor Synergistic Therapy by AR‐ZS/ID‐P NPs

Encouraged by the excellent in vitro and in vivo targeting ability of AR‐ZS/ID‐P NPs, we further investigated their anti‐tumor synergistic therapeutic effects in vivo. All mice were sacrificed 14 days post‐treatment, and weight of excised tumors were shown in Figure 6B. It is clear that, among all groups, the group of AR‐ZS/ID‐P NPs with laser irradiation showed the best tumor inhibition efficacy. The smallest tumor weight was obtained in the group treated with AR‐ZS/ID‐P NPs with laser irradiation, which was only about 25% of the PBS group's samples. The posttreatment tumor volume also indicated that AR‐ZS/ID‐P NPs with laser irradiation inhibited tumor growth rapidly and displayed a remarkable tumor inhibition effect after 14 days (Figure 6C). Representative photographs of mice 14 days after various treatments further confirmed their photothermal therapy effects (Figure S9, Supporting Information). The scars caused by PTT were visible in the AR‐ZS/ID‐P NPs and AR‐ZS/I‐P NPs groups, whereas a smaller scar was detected in the ZS/ID‐P NPs group, and no scar was observed in other groups. Moreover, no noticeable change in body weight was observed for all mice in the six groups, indicating hardly any systemic toxicity of all samples and laser irradiation (Figure 6D). The histological sections of the tumor were studied by hematoxylin and eosin (H&E) staining. In Figure 6E, sliced tumors exhibited massive cell necrosis and apoptosis under the treatment of NIR‐mediated AR‐ZS/ID‐P NPs. In contrast, nearly no obvious tumor cell necrosis was observed in the PBS group, and the remaining groups only showed partial necrosis. These optimistic effects of the group of AR‐ZS/ID‐P NPs with laser irradiation on tumor inhibition might be due to three factors. First, the AR peptide helps the AR‐ZS/ID‐P NPs home to the tumor sites. Second, more AR‐ZS/ID‐P NPs enter MCF‐7 cancer cells leading to an enhanced chemotherapy effect. Third, 808 nm NIR irradiation treatment triggers PDT/PTT effects, further inhibiting tumor growth.

Additionally, no remarkable lesions were found in major healthy organs under all treatments, highlighting the in vivo biosafety of using our NPs for cancer treatment (Figure S10, Supporting Information). Furthermore, the liver index and blood chemistry (including ALT, AST, ALP, LDH, and BUN) of the mice treated with ZIF‐8, free ID, and AR‐ZS/ID‐P NPs showed no differences from the PBS group (Figure S11, Supporting Information). Overall, the results confirmed that AR‐ZS/ID‐P NPs were safe and could serve as a nanocarrier for drug delivery and cancer therapy.

## 3 Conclusion

In summary, we have developed an ideal tumor‐targeted and pH‐responsive nanoplatform, AR‐ZS/ID‐P NPs, for the integration of PTT/PDT and chemotherapy for synergistic cancer treatment. By adjusting the concentration of the SF bio‐template, we achieved AR‐ZS/ID‐P NPs with reasonable size and good dispersity, and high encapsulation efficiency of both ICG and DOX. The pH‐sensitive behavior of ZIF‐8 was maintained in AR‐ZS/ID‐P NPs, allowing for triggered drug release in the acidic tumor microenvironment but remaining stable in neutral conditions. With AR peptide modification, AR‐ZS/ID‐P NPs were selectively homed and recruited to tumor cells. Under laser irradiation, ICG produced destructive heat and ROS to achieve PTT and PDT and enabled thermal and fluorescence imaging that can further guide DOX‐triggered chemotherapy. In vivo experiments confirmed enhanced tumor accumulation of AR‐ZS/ID‐P NPs and significant tumor growth inhibition. More importantly, AR‐ZS/ID‐P NPs exhibited good biocompatibility and reduced systemic toxicity. Collectively, AR‐ZS/ID‐P NPs represent a promising multifunctional carrier for superior tumor therapy with opportunities for significant clinical applications.

## 4 Experimental Section

### Formation of SF Mediated ZIF‐8 Nanoparticles (ZS NPs)

The silk fibroin (SF) was extracted from *Bombyx mori* cocoons and converted into an SF aqueous solution following a previous protocol.^[^ ^16^ ^]^ To explore the impact of SF concentrations on the formation of ZS NPs, the SF aqueous solutions were adjusted to different concentrations (0.5%, 1.0%, and 2.0%) using deionized water (DI water). An FITC‐labeled SF aqueous solution (f@SF) was also prepared using a final concentration of 1 mg FITC/10 mg protein. The mixture was shaken at a speed of 200 rpm at 25 °C overnight, put into a dialysis bag (8000–14 000 Da), and dialyzed against DI water for 3 days to remove any unreacted FITC. To prepare ZS NPs, 3 mL of each concentration of the SF aqueous solution (with DI water as the control) was misxed with an equal volume of a stock solution of 2‐HmIm (1600 mm) separately. Then, 3 mL of a 44.44 mm Zn(NO_3_)_2_·6H_2_O aqueous solution was added to the well‐mixed solution, followed by thorough shaking at 37 °C. The formation mechanism was investigated by collecting 10 µL of samples from the corresponding medium at 0.5, 1, 2, 4, 6, 8, 10, and 15 min. Finally, the ZS NPs were collected after being washed with DI water three times and resuspended in DI water for further tests. The f@SF aqueous solution was used with a concentration of 10 mg mL^−1^ to prepare FITC‐labeled ZS NPs (f@ZS NPs) using the same method as for the ZS NPs.

### Synthesis of DOX and ICG Loaded ZS NPs (ZS/ID NPs)

A one‐pot process was employed to prepare ZS/ID NPs. DOX and ICG were diluted to various concentrations (0.5, 1.0, 2.0, and 4.0 mg mL^−1^) using DI water. One milliliter of SF aqueous solution (30 mg mL^−1^) was misxed with 3 mL of 2‐HmIm aqueous solution (1600 mm), followed by the addition of 1 mL of DOX stock solution, 1 mL of ICG stock solution, and 3 mL of Zn(NO_3_)_2_·6H_2_O aqueous solution (44.44 mm). The resulting mixture was kept for 15 min shaking at 37 °C to facilitate the formation of ZS/ID NPs. The products were then rinsed with DI water until the supernatants were clear. And the supernatants were collected to calculate the drug encapsulation efficiency (EE). The purified ZS/ID NPs were freeze‐dried to form ZS/ID NP powders. The EE was assessed by the following formula. The absorbance of DOX and ICG in the supernatants was measured at 490 and 780 nm, respectively. A standard curve was used to calculate the mass of DOX according to the absorbance.

(1) $$\mathrm{EE}\;\left(\%\right)\;=\;\left(\mathrm{Mt}-\mathrm{Ms}\right)\;/\mathrm{Mt}\times \;100\%$$

where Mt and Ms were the total mass of DOX or ICG added and the mass of DOX or ICG in the supernatant.

### Preparation of PEI Coated and Peptide Conjugated Tumor‐Homing ZS/ID NPs (AR‐ZS/ID‐P NPs)

To enhance the stability of nanoparticles and increase the binding sites for MCF‐7 breast tumor‐targeting peptide (AR), a PEI coating strategy was employed for both ZS/ID NPs and f@ZS NPs. The ZS/ID NPs and f@ZS NPs powders were suspended in a PBS solution with a concentration of 5 mg mL^−1^, and then co‐incubated with PEI solution (10 000 Da, 5 mg mL^−1^) under mild shaking at 37 °C. After 1 h, the PEI‐coated ZS/ID NPs (ZS/ID‐P NPs) and f@ZS NPs (f@ZS‐P NPs) were obtained by centrifugation, and excess PEI was washed out. The purified ZS/ID‐P NPs and f@ZS‐P NPs were adjusted to 5 mg mL^−1^ using DI water, and both types of nanoparticles were rich in –NH_2_ due to the PEI coating. The targeted AR peptide and RGD peptide (control group) were synthesized by GL Biochem Ltd. (Shanghai, China). Stock solutions of peptides were prepared at 1 mg mL^−1^ in PBS. To conjugate the peptides, EDC (16 mg) and NHS (4 mg) were added to 3 mL of peptide stock solution to activate ‐COOH groups at room temperature for 15 min. Then, 3 mL of PEI‐coated nanoparticles were added to the carboxylated peptide stock solution and left on a rocker shaker for 2 h at room temperature. After conjugation, a series of centrifugation and washing steps were performed to remove any unbound peptide. Using this method, all of the peptide‐conjugated nanoparticles, including AR‐ZS/ID‐P NPs, RGD‐ZS/ID‐P NPs, AR‐f@ZS‐P NPs, and RGD‐f@ZS‐P NPs, were synthesized.

### Characterization of Nanoparticles

The morphology of the nanoparticles was examined using scanning electron microscopy (SEM) and transmission electron microscopy (TEM). The size, surface zeta potential, and dispersibility of the nanoparticles were analyzed using a Nano Particle Analyzer with dynamic light scattering (DLS). The absorption spectra of the nanoparticles were measured using a microplate assay to confirm the presence of ICG and DOX within the range of 400 to 900 nm. Prior to analysis, all samples were diluted with DI water and dispersed evenly by sonication. Additionally, Powder X‐ray diffraction (PXRD) and Fourier transform infrared spectroscopy (FTIR) analyses were conducted to reveal the microstructure and phase composition of the nanoparticles.

### Drug Release Behavior of AR‐ZS/ID‐P NPs

AR‐ZS/ID‐P NPs were suspended in PBS solution (1 mg mL^−1^, pH 5.0, 6.4, 7.4). The release efficiency was tested by collecting 1 mL of sample from the corresponding medium followed by adding 1 mL of the fresh medium at 0.5, 1, 2, 4, 6, 8, 10, 12, and 24 h. The collected solutions were centrifuged to obtain the supernatant used for determining the absorbance at 490 nm for DOX or 780 nm for ICG. According to the absorbance, the mass of DOX or ICG was calculated through a standard curve, and the following formula was used to calculate the release percentage of DOX or ICG:

(2) $$\mathrm{Release}\mathrm{percentage}\;\left(\%\right)=\mathrm{Mr}/\mathrm{Ml}\times 100\%$$

where Mr and Ml were the total mass of DOX or ICG released and loaded.

After incubation for 24 h, the samples treated with different pH values were drop‐casted onto silicon substrates and air‐dried at room temperature. The morphology of the samples was observed using SEM.

### Photothermal/Photodynamic Abilities of AR‐ZS/ID‐P NPs

The photothermal and photodynamic abilities of AR‐ZS/ID‐P NPs may be influenced by ICG concentration and laser power density. To investigate the impact of ICG concentration on their photothermal abilities, aqueous solutions of PBS and AR‐ZS/ID‐P NPs (3 mL) with different ICG concentrations (10, 20, and 30 µg mL^−1^) were irradiated with an 808 nm continuous laser at a power density of 2 W cm^−2^. Laser power densities of 0.5, 1, and 2 W cm^−2^ at 808 nm were introduced to explore their effects on AR‐ZS/ID‐P NPs (30 µg mL^−1^). To compare the photothermal ability of different nanoparticles, aqueous solutions of AR‐ZS/ID‐P NPs, free ICG with the same ICG concentration (30 µg mL^−1^), and ZIF‐8 (used as a control) were irradiated with an 808 nm continuous laser at 2 W cm^−2^. All samples were irradiated for 10 min, and their temperatures were monitored every minute by Infrared Thermal Imaging Spectrometer.

The photothermal conversion efficiency of ICG or AR‐ZS/ID‐P NPs was calculated using the following formula described in previous studies:^[^ ^28^ ^]^

(3) $$\eta \;=\frac{hA\left(\Delta T_{max,\;mix}-\Delta T_{max,\;PBS}\right)}{I\left(1-10^{-A_{808}}\right)}\;$$

where η is the thermal conversion efficiency. ∆*T*_max,min_ and ∆*T*_max,PBS_ were determined from the temperature changes between steady conditions and irradiation treatment. *I* was also clear for it was the product by multiplying irradiation power 2 W cm^−2^ and the exposed area of the solution. *A*_808_ is the absorption at 808 nm of AR‐ZS/ID‐P NPs. The only unknown *h*A could be calculated by introducing *θ*, which came from the ratio of ∆*T* and ∆*T*_max_, so as to use the linear relationship between time and −ln*θ* of the cooling stage. Then, each known quantity was substituted into the formula, and finally the photothermal conversion efficiency was obtained.

To assess the photodynamic ability of AR‐ZS/ID‐P NPs, 1,3‐Diphenylisobenzofuran (DPBF) was used as an indicator. DPBF can react irreversibly with ^1^O_2_, resulting in a decrease in its absorbance. DPBF in ethanol (100 µL, 5 mm) was added to an aqueous solution of AR‐ZS/ID‐P NPs (30 µg mL^−1^ ICG, 5 mL). The solution was then irradiated with an 808 nm laser (2 W cm^−2^), and 150 µL of the sample was collected at 0, 1, 2, 3, 4, 5, 6, 8, 10, and 15 min. After centrifugation, the supernatant solution of each sample was added to a 96‐well plate, and their absorption spectra from 350 to 900 nm were recorded using a microplate reader.

### AR‐f@ZS‐P NPs Targeting Effect In Vitro

To investigate the in vitro targeting effect of AR‐f@ZS‐P NPs, MCF‐7 cells were seeded onto coverslips in complete medium (DMEM with 10% fetal bovine serum and 1% Penicillin‐Streptomycin) at a concentration of ≈2.5 × 10^5^ cells per well (37 °C, 5% CO_2_). After 24 h, the culture medium was replaced with fresh medium containing f@ZS‐P NPs, RGD‐f@ZS‐P NPs, or AR‐f@ZS‐P NPs (100 µg mL^−1^ for each), and co‐cultured for an additional 6 h. The medium was then removed, and the plates were washed with PBS to remove any free nanoparticles. The cells were fixed with 4% paraformaldehyde in PBS for 15 min and stained with 4′,6‐diamidino‐2‐phenylindole (DAPI). Finally, all cells were imaged using a confocal laser scanning microscope (CLSM).

### Cytotoxicity and Cellular Uptake of AR‐ZS/ID‐P NPs In Vitro

To evaluate cytotoxicity, MCF‐7 cells were seeded at a density of 1×10^5^ cells per well in a 96‐well plate and cultured with complete medium at 37 °C and 5% CO_2_. After 24 h, the treated medium was removed, and ZS NPs, ZS/I NPs, ZS/D NPs, ZS/ID NPs, AR‐ZS/ID‐P NPs (each at concentrations of 0, 20, 40, 80, or 100 µg mL^−1^), or free ICG/DOX (termed ID, at concentrations of 0, 5, 10, 20, or 25 µg mL^−1^) in fresh complete medium were added to the cells to compare the chemotherapeutic efficacy. Meanwhile, to confirm the photothermal/photodynamic abilities of ICG‐loaded nanoparticles, cells treated with ZS/ID NPs and AR‐ZS/ID‐P NPs (each at a concentration of 0, 20, 40, 80, or 100 µg mL^−1^) were irradiated with an 808 nm (2 W cm^−2^) laser for 5 min; free ID (at a concentration of 0, 5, 10, 20, or 25 µg mL^−1^) with the same treatment was used as a positive control. All treated samples were discarded, and the cells were washed twice with PBS 12 h later. Then, the Cell Counting Kit‐8 (CCK‐8) was used to estimate cell proliferation and cytotoxicity. Briefly, 100 µL of fresh culture medium, along with 10 µL of CCK‐8, were added to every well. The absorbance of CCK‐8 at 450 nm was measured after 2 h. Calcein AM/PI staining was used to stain the live and dead cells, followed by imaging under CLSM. DCFH‐DA was employed as an intracellular ROS probe to observe intracellular ROS generation under different treatments. In detail, MCF‐7 cells were seeded in the six‐well culture plates (5×10^4^ cells per well) and cultured overnight. Then the fresh culture media containing ZS NPs, free ID, ZS/I NPs, ZS/ID NPs, or AR‐ZS/ID‐P NPs was added. After 4 h, the cells were washed twice, and then added with 1 mL of DCFH‐DA diluent. After another 30 min, the cells were washed again and irradiated with 808 nm laser (2.0 W cm^−2^, 5 min). Finally, the green emission of these treated cells was imaged by CLSM.

The cellular uptake of nanoparticles was also assessed by CLSM using the same method as the targeting ability study. In brief, free ID, ZS/ID NPs, and AR‐ZS/ID‐P NPs, all with the same ICG concentration (6 µg mL^−1^), were incubated with MCF‐7 cells for 1 and 12 h, then washed twice with cold PBS. The cells were then fixed and stained, followed by imaging under CLSM. Image J was used to calculate the fluorescence intensity of MCF‐7 cells. In addition, TEM was used to observe the AR‐ZS/ID‐P NPs‐treated cells.

### Construction of MCF‐7 Tumor Models of Mice

The female athymic BALB/c nude mice (20–25 g) were purchased from Shanghai Laboratory Animal Center and raised in the Laboratory Animal Center of Zhejiang University. The MCF‐7 tumor mice models were constructed by injecting 5 × 10^6^ cells into the dorsal skin of the mice and used for the subsequent experiments until the tumor reached ≈100 mm^3^. All animal studies were approved by Animal Ethics Committee of Zhejiang University.

### Targeting Ability and Photothermal Imaging of the NPs In Vivo

For in vivo near‐infrared ray (NIR) fluorescence images, 15 mice were separated into 3 groups (free ID, ZS/ID NPs, and AR‐ZS/ID‐P NPs, *n* = 5). One hundred microliters of sample solution with an equal ICG concentration of 60 µg mL^−1^ was injected into the caudal vein of corresponding mice. All mice were given anesthesia using a 4% isoflurane/oxygen mixture and scanned under the In vivo Xtreme Imaging System with an excitation of 710 nm at 1, 4, 8, 12, and 24 h after injection. The tumor and other major organs (heart, lungs, spleen, kidney, and liver) were excised and captured in fluorescence images in the same way. Image J was used to calculate the bioluminescence signal intensity of ex vivo tissues.

Similar to NIR fluorescence imaging, 20 mice were separated into 4 groups (free ID, ZS/ID NPs, AR‐ZS/I‐P NPs, and AR‐ZS/ID‐P NPs, *n* = 5) for IR thermal imaging. One hundred micoliters of sample solution with an equal ICG concentration of 60 µg mL^−1^ was injected into the caudal vein of corresponding mice. After 4 h, the 808 nm laser (2 W cm^−2^) was irradiated on the tumor site for 5 min, and the Infrared Thermal Imaging Spectrometer was used for IR thermal imaging.

### Antitumor Effect of AR‐ZS/ID‐P NPs In Vivo

To evaluate the in vivo anti‐tumor effect of AR‐ZS/ID‐P NPs, six groups of mouse models (*n* = 5) were subjected to different treatments: 1) AR‐ZS/ID‐P NPs + laser; 2) AR‐ZS/I‐P NPs + laser; 3) AR‐ZS/D‐P NPs; 4) ZS/ID‐P NPs + laser; 5) free ID + laser, and 6) PBS. Groups 1–5 were adjusted to the same concentration of ICG or DOX (60 µg mL^−1^). A volume of 100 µL of the samples was intratumorally injected into the mice four times with an interval of 3 days. Four hours after injection, the laser‐treated groups were irradiated with an 808 nm (2 W cm^−2^) laser for 10 min, and the temperature change of the tumors was monitored by an Infrared Thermal Imaging Spectrometer. The weight of the mice and the volume of the tumors were measured every 2 days. After 14 days from the first injection, the mice were sacrificed, and the tumors and major organs (heart, lungs, liver, kidney, and spleen) were excised. The excised tumors were weighed, and all excised tumors and major organs were sectioned for Hematoxylin‐eosin (H&E) staining.

The safety of AR‐ZS/ID‐P NPs was also evaluated in vivo. Twenty female athymic BALB/c nude mice were divided into four groups (*n* = 5). A volume of 100 µL of ZIF‐8, ZS/ID‐P NPs, and AR‐ZS/ID‐P NPs each at 1.0 mg mL^−1^ was injected intravenously into the corresponding mice every 4 days. PBS was used as a control. After the last injection, all the mice were fasted. Twenty‐four hours later, the mice were anesthetized, and blood was collected from their eyeballs. The collected blood was allowed to stand for at least 12 h at 4 °C, followed by centrifugation at 3000 rpm for 5 min to obtain serum. The levels of ALT, AST, ALP, LDH, and BUN in serum were assayed. The liver was removed from the euthanized mice and the liver index was determined according to the following formula:

(4) $$\begin{array}{c} \text{Liver index}=(\text{Weight of the liver/Weight of the experimental mice})/\\ \left(\text{weight of the control liver/Weight of the control mice}\right) \end{array}$$

### Statistical Analysis

Data analysis was performed using SPSS. One‐way analysis of variance (ANOVA) was used to analyze inter‐group differences. Results were presented as means ± standard deviation and considered significant or highly significant at *p < 0.05 and **p < 0.01, respectively.

## Conflict of Interest

The authors declare no conflict of interest.

## Supporting information

Supporting Information

## Data Availability

The data that support the findings of this study are available from the corresponding author upon reasonable request.
