# Supplementary material for: Metal−Organic Frameworks Nucleated by Silk Fibroin and Modified with Tumor‐Targeting Peptides for Targeted Multimodal Cancer Therapy
Source: Adv Sci (Weinh). 2023 Aug 23;10(28):2302700. doi: 10.1002/advs.202302700 (PMC10558676; doi:10.1002/advs.202302700)
Supplement: Supplementary file 1 — Supporting Information [file ADVS-10-2302700-s001.pdf]

ADVANCED SCIENCE
Open Access

Supporting Information

for *Adv. Sci.*, DOI 10.1002/advs.202302700

Metal–Organic Frameworks Nucleated by Silk Fibroin and Modified with Tumor-Targeting Peptides for Targeted Multimodal Cancer Therapy

Yuping Chen, Ruyin Lyu, Jie Wang, Qichao Cheng, Yanfang Yu, Shuxu Yang, Chuanbin Mao* and Mingying Yang**

Supporting Information

Metal−Organic Frameworks Nucleated by Silk Fibroin and Modified with Tumor-Targeting Peptides for Targeted Multimodal Cancer Therapy

Yuping Chen, Ruyin Lyu, Jie Wang, Qichao Cheng, Yanfang Yu, Shuxu Yang, Chuanbin Mao* and Mingying Yang**

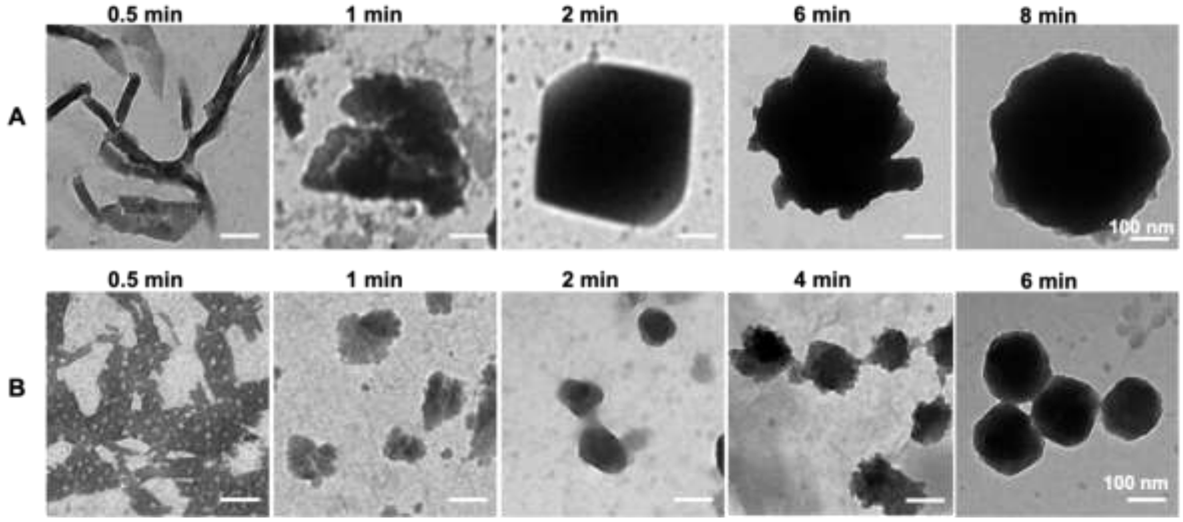

Figure S1. The formation process of ZIF-8 and ZS NPs. A) TEM images of ZIF-8 at 0.5 min, 1 min, 2 min, 6 min, and 8 min. B) TEM images of ZS NPs at 0.5 min, 1 min, 2 min, 4 min, and 6 min. The differences between ZIF-8 and ZS NPs could be observed within 0.5 min. The ZS NPs group formed inerratic rods at about 100-150 nm, whereas the ZIF-8 group formed longer rods (200-300 nm). This might result in their difference in the final size. The final size of ZIF-8 and ZS NPs was about 400 nm and 150 nm, respectively.

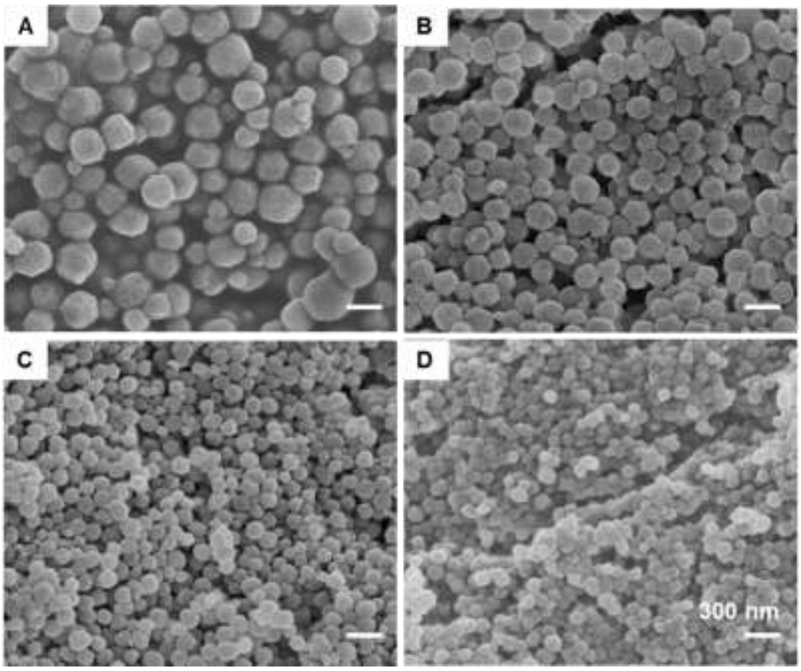

Figure S2. SEM images of ZS/ID NPs with added ID of different concentrations: A) 0.5 mg/mL, B) 1.0 mg/mL, C) 2.0 mg/mL and D) 4.0 mg/mL. An equal amount of ICG and DOX were mixed to form ID.

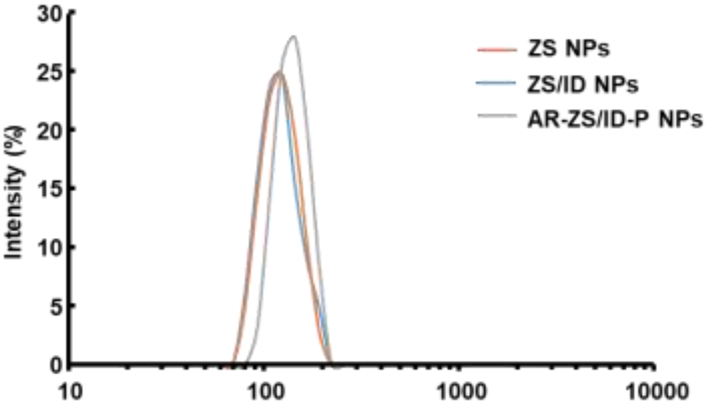

Figure S3. The size distribution of ZS NPs, ZS/ID NPs and AR-ZS/ID-P NPs obtained from DLS analysis.

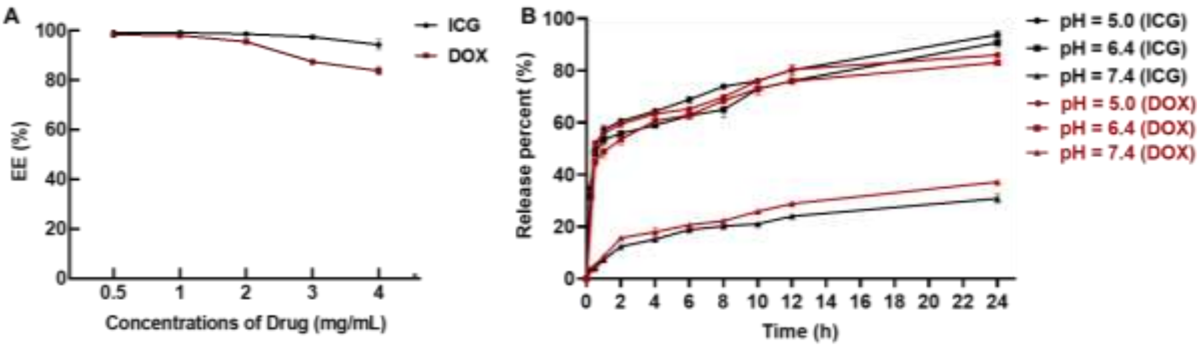

Figure S4. Loading and release behaviors of AR-ZS/ID-P NPs. A) Encapsulation efficiency (EE) of DOX and ICG onto AR-ZS/ID-P NPs with various concentrations, respectively. B) The DOX and ICG released in acidic (pH = 5.0, pH = 6.4) and neutral (pH = 7.4) medium from AR-ZS/ID-P NPs, indicating the controlled DOX and ICG release from AR-ZS/ID-P NPs selectively under acidic pH.

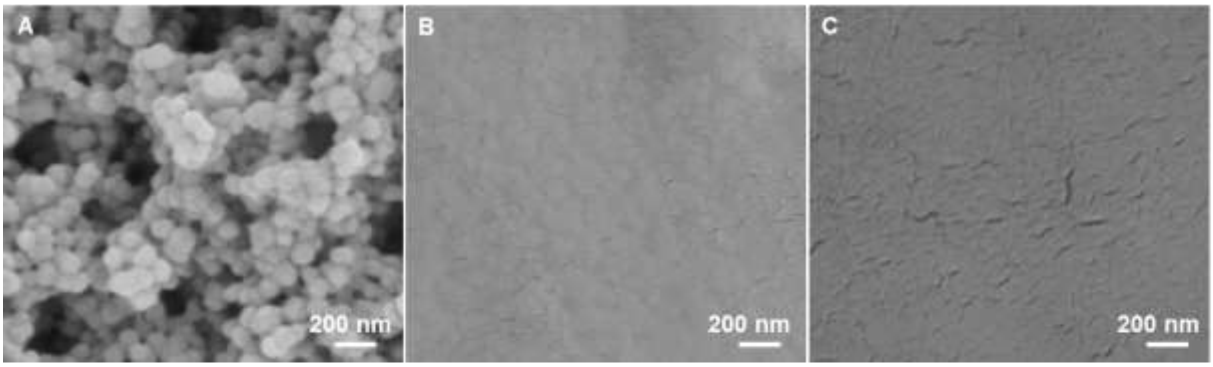

Figure S5. SEM images of AR-ZS/ID-P NPs incubated in different pH solutions for 24 hours: A) pH = 7.4; B) pH = 6.4; C) pH = 5.0.

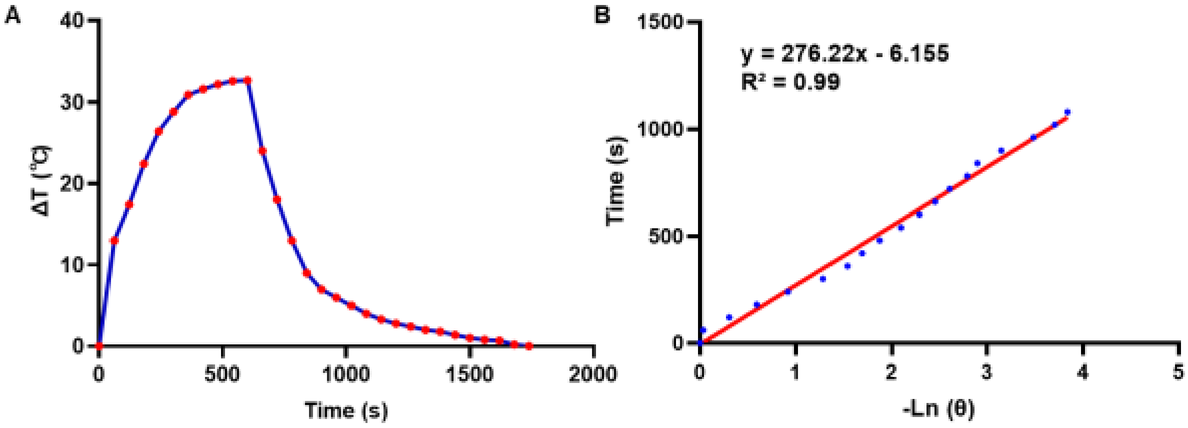

Figure S6. The photothermal conversion efficiency of AR-ZS/ID-P NPs. A) Temperature increase profile of AR-ZS/ID-P NPs solution with laser irradiation and cooling profile without irradiation. B) Linear profile of time versus -Ln(θ) obtained from the cooling period in A).

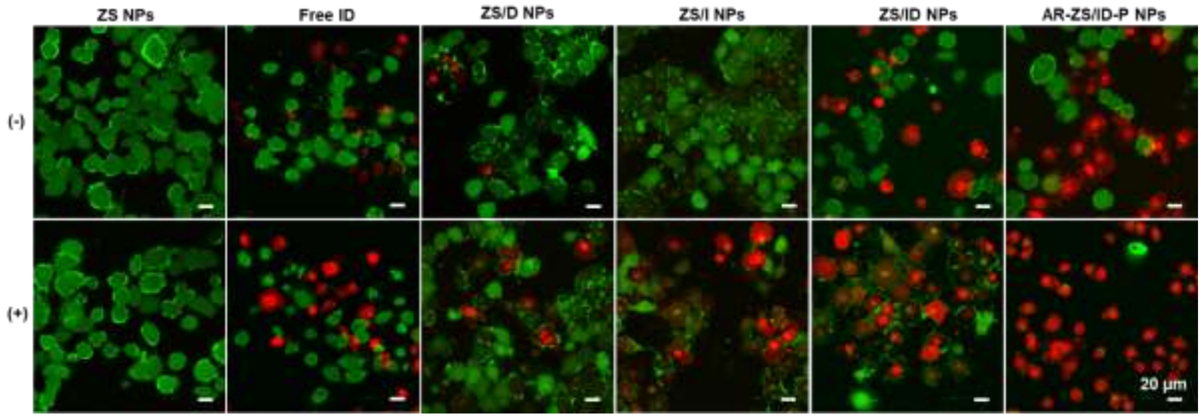

Figure S7. Fluorescence live/dead cell images of MCF-7 cells at different treatment groups. (+): with 808 nm laser irradiation; (-): without laser irradiation.

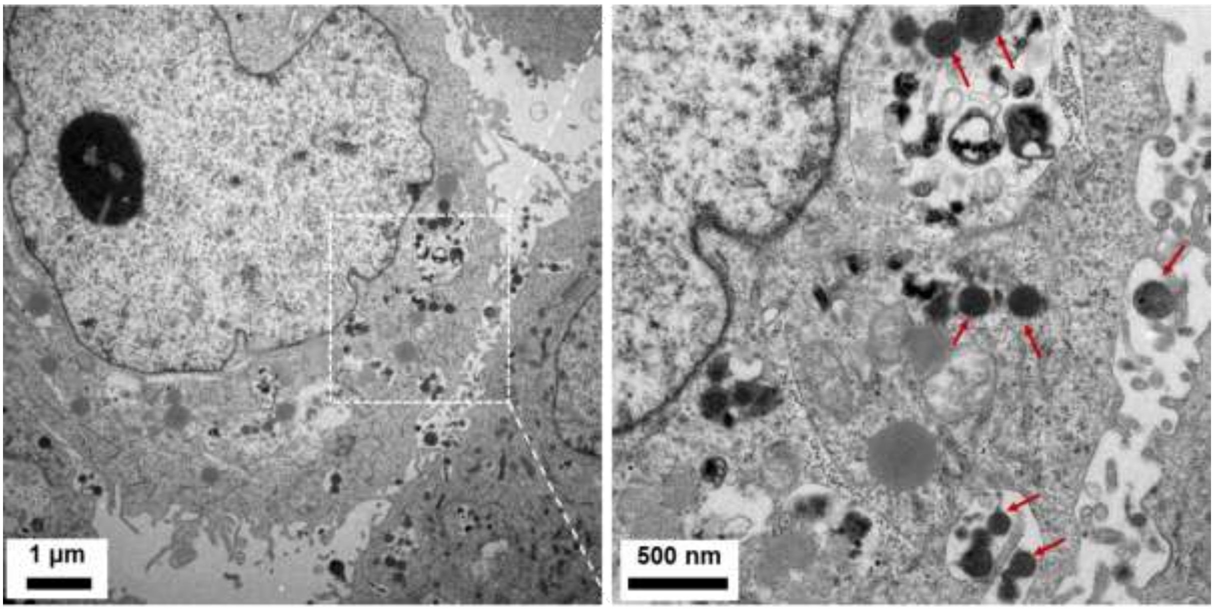

Figure S8. TEM images of MCF-7 cells co-cultured with AR-ZS/ID-P NPs (100 μg/mL) for 6 h. Red arrows showed the internalization of AR-ZS/ID-P NPs into MCF-7 cells. The right image is the magnified portion in the left image highlighted by white frames.

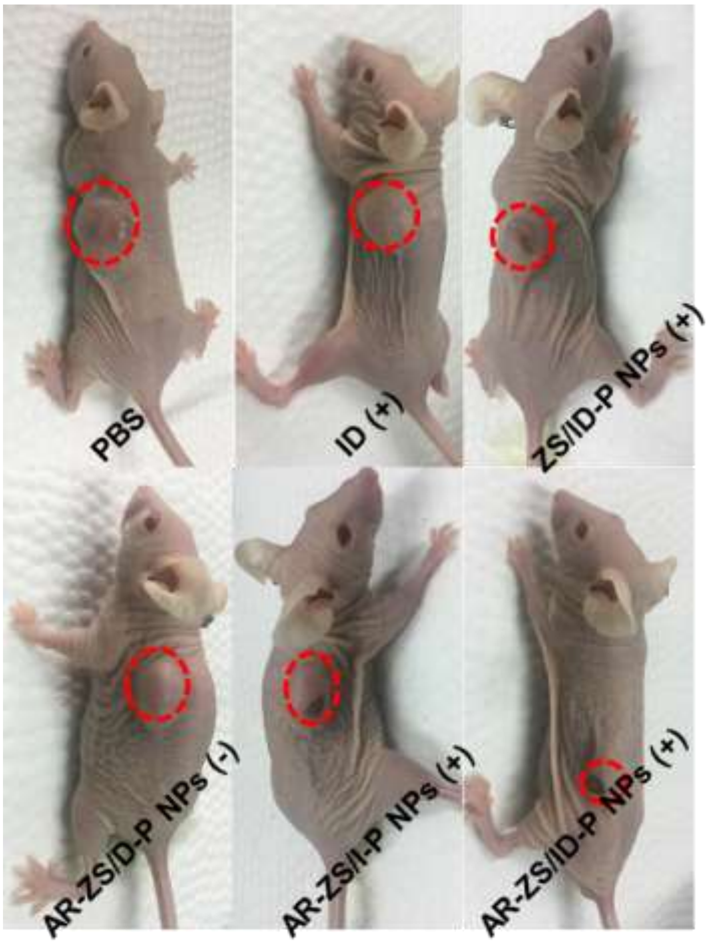

Figure S9. Representative photographs of mice after different treatments indicated. The subcutaneous tumors are marked with red dotted circle. (+): with 808 nm laser irradiation; (-): without laser irradiation.

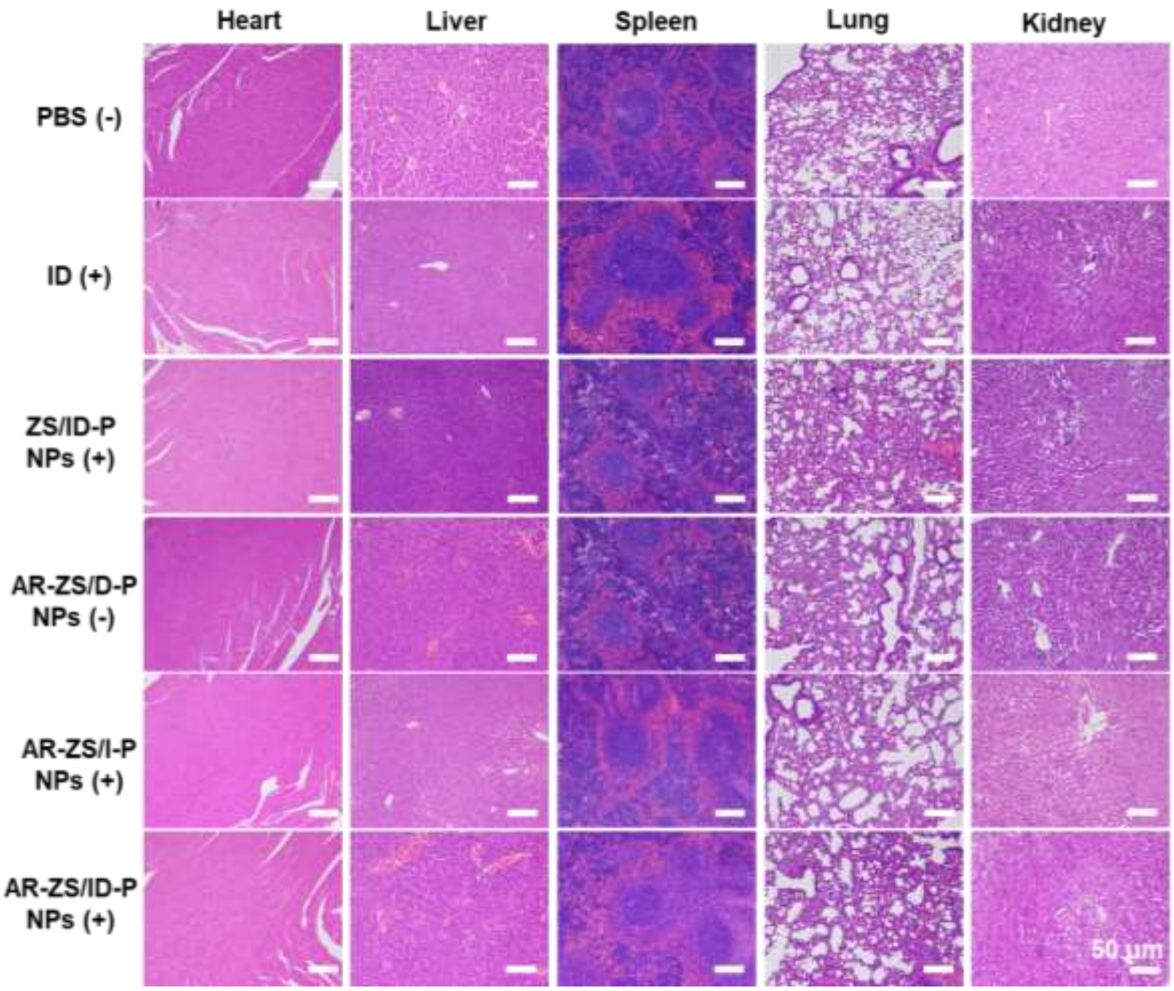

Figure S10. H&E-staining of major organs (heart, spleen, lung, liver, and kidney) collected from the MCF-7 tumor-bearing mice treated by intravenous injection of PBS, ID , ZS/ID-P NPs, AR-ZS/D-P NPs. "(-)" and "(+)" means single injection therapy and combined laser treatment, respectively.

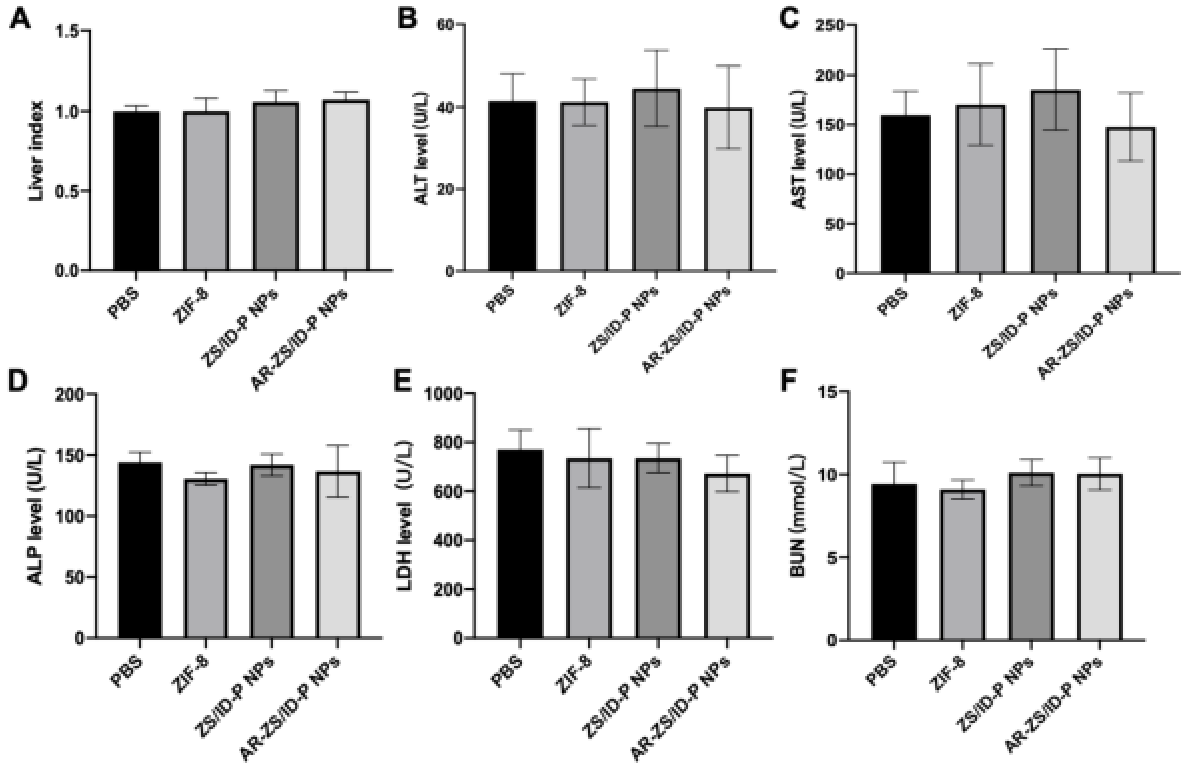

Figure S11. Biocompatibility of AR-ZS/ID-P NPs in vivo. (A) Liver index of the mice under the treatments of PBS, ZIF-8, ZS/ID-P NPs, and AR-ZS/ID-P NPs. (B-F) Analysis of biochemical alterations (ALT, AST, ALP, LDH, and BUN) induced PBS, ZIF-8, ZS/ID-P NPs, and AR-ZS/ID-P NPs. (mean ± SD, n = 5, *$p < 0.05$; **$p < 0.01$).

Table S1. The polydispersity index of ZS NPs, ZS/ID NPs and AR-ZS/ID-P NPs.

	**Diameter (nm) Mean ± SD**	**Polydispersity Index (PDI)**
**ZS NPs**	122.01 ± 6.36	0.114
**ZS/ID NPs**	120.71 ± 2.45	0.101
**AR-ZS/ID-P NPs**	135.45 ± 1.89	0.086
